# Cartilage Endplate Stem Cells Transdifferentiate Into Nucleus Pulposus Cells via Autocrine Exosomes

**DOI:** 10.3389/fcell.2021.648201

**Published:** 2021-03-04

**Authors:** Liwen Luo, Junfeng Gong, Hongyu Zhang, Jinghao Qin, Changqing Li, Junfeng Zhang, Yu Tang, Yang Zhang, Jian Chen, Yue Zhou, Zhiqiang Tian, Yao Liu, MingHan Liu

**Affiliations:** ^1^Department of Orthopaedics, Xinqiao Hospital, Army Medical University, Third Military Medical University, Chongqing, China; ^2^Institute of Immunology, PLA, Army Medical University, Third Military Medical University, Chongqing, China; ^3^Department of Emergency, Second Affiliated Hospital of Chongqing Medical University, Chongqing, China; ^4^Institute of Hepatopancreatobiliary Surgery, Chongqing General Hospital, University of Chinese Academy of Sciences, Chongqing, China; ^5^State Key Laboratory of Silkworm Genome Biology, Biological Science Research Center, Southwest University, Chongqing, China; ^6^Department of Pharmacy, Daping Hospital, Army Medical University, Third Military Medical University, Chongqing, China

**Keywords:** cartilage endplate stem cells, differentiation, exosome, GATA binding protein 4, transforming growth factor-β1, intervertebral disk degeneration

## Abstract

Stem cells derived from cartilage endplate (CEP) cells (CESCs) repair intervertebral disc (IVD) injury; however, the mechanism remains unclear. Here, we evaluated whether CESCs could transdifferentiate into nucleus pulposus cells (NPCs) via autocrine exosomes and subsequently inhibit IVD degeneration. Exosomes derived from CESCs (CESC-Exos) were extracted and identified by ultra-high-speed centrifugation and transmission electron microscopy. The effects of exosomes on the invasion, migration, and differentiation of CESCs were assessed. The exosome-activating hypoxia-inducible factor (HIF)-1α/Wnt pathway was investigated using lenti-HIF-1α and Wnt agonists/inhibitors in cells and gene ontology and Kyoto Encyclopedia of Genes and Genomes enrichment analysis in normal and degenerated human CEP tissue. The effects of GATA binding protein 4 (GATA4) on transforming growth factor (TGF)-β expression and on the invasion, migration, and transdifferentiation of CESCs were investigated using lenti-GATA4, TGF-β agonists, and inhibitors. Additionally, IVD repair was investigated by injecting CESCs overexpressing GATA4 into rats. The results indicated that CESC-Exos promoted the invasion, migration, and differentiation of CESCs by autocrine exosomes via the HIF-1α/Wnt pathway. Additionally, increased HIF-1α enhanced the activation of Wnt signaling and activated GATA4 expression. GATA4 effectively promoted TGF-β secretion and enhanced the invasion, migration, and transdifferentiation of CESCs into NPCs, resulting in promotion of rat IVD repair. CESCs were also converted into NPCs as endplate degeneration progressed in human samples. Overall, we found that CESC-Exos activated HIF-1α/Wnt signaling via autocrine mechanisms to increase the expression of GATA4 and TGF-β1, thereby promoting the migration of CESCs into the IVD and the transformation of CESCs into NPCs and inhibiting IVDD.

## Introduction

Low back pain (LBP) is a pathology that can cause disability, leading to increased social burden among the expanding and aging population (GBD 2016 Disease and Injury Incidence and Prevalence Collaborators, 2017), and intervertebral disc (IVD) degeneration (IVDD) is the most common cause of LBP (Freemont, 2009; Schol and Sakai, 2019). IVDD is an age-related disease associated with multiple factors (Stokes and Iatridis, 2004; Risbud and Shapiro, 2014; Vo et al., 2016) and characterized by the degradation of extracellular matrix components, such as collagens, proteoglycans, and fibronectin/laminins, as well as upregulation of matrix-degrading enzymes (Le Maitre et al., 2004; Roughley, 2004). Nucleus pulposus (NP) and annulus fibrosus (AF) are the main structural elements of IVDs. NP cells (NPCs) secrete extracellular matrix components to maintain the biomechanical functions of the IVD, and hypocellularity of functional NP cells leads to IVDD (Vo et al., 2016). Thus, regenerating NPCs could be a potential therapeutic strategy for IVDD (Li et al., 2017; Zhang et al., 2020). In clinical trials, autologous or allogeneic mesenchymal stem cell (MSC) transplantation has been performed in IVD repair and LBP release (Orozco et al., 2011; Noriega et al., 2017). In our previous study, we identified cartilage endplate (CEP) stem cells (CESCs) in the CEP (Liu et al., 2011). These CESCs were found to be similar to MSCs in terms of stem cell characteristics; however, they showed better capacity in osteogenesis and chondrogenesis. Additionally, CESCs with the characteristics of stem cells in CEP tissues have been shown to migrate to the NP and differentiate into NPCs, thereby promoting the repair of the IVD and inhibiting IVDD (Wang et al., 2014). However, the specific mechanisms remain unclear.

Exosomes are extracellular vesicles of endosomal origin that are secreted by prokaryotes and eukaryotes (Kalluri and Lebleu, 2020) and exhibit a diameter of 40–160 nm (Kourembanas, 2015). These vesicles are present in a variety of body fluids, including plasma, semen, saliva, urine, amniotic fluid, synovial fluid, and breast milk (Simpson et al., 2008; Properzi et al., 2013). Exosomes have been shown to encapsulate many different components, including mRNAs, microRNAs, ribosomal RNAs, non-coding RNAs, cytokines, proteins, and lipids (Simons and Raposo, 2009). Additionally, exosomes play important roles in altering signaling transduction related to proliferation, differentiation, autophagy, and other cellular activities (Barile and Vassalli, 2017; Chang et al., 2018). Notably, MSC-derived exosomes can ameliorate IVDD via various mechanisms after injection into the IVD (Liao et al., 2019; Xia et al., 2019). Moreover, previous studies have shown that CESCs can inhibit the apoptosis of NPCs by secreting exosomes and thus slow down or reverse the IVDD process (Luo et al., 2021). However, we do not know whether the migration and differentiation of CESCs into NPCs can be regulated by CESC-Exos in an autocrine manner, thereby slowing down IVDD; this mechanism could provide an innovative approach to the treatment of IVDD by regulating CESC migration and differentiation.

GATA-binding protein 4 (GATA4), a DNA-binding zinc finger transcription factor, has been shown to regulate various biological processes, including proliferation, differentiation, and angiogenesis (Molkentin, 2000; Patient and Mcghee, 2002). During bone formation and development, GATA4 plays important roles in promoting osteogenic differentiation and function by regulating Runt-related transcription factor 2, bone morphogenic protein, transforming growth factor (TGF)-β, Fas ligand, and other key proteins (Miranda-Carboni et al., 2011; Güemes et al., 2014; Khalid et al., 2018). Recent studies have shown that GATA4, a key transcription factor, regulates MSC differentiation (Li et al., 2015; Xu et al., 2016). However, to date, no researchers have evaluated the roles of GATA4 in the migration and differentiation of CESCs into NPCs.

Accordingly, in this study, we assessed whether autocrine exosomes could promote CESC migration and transdifferentiation into NPCs by activating the hypoxia-inducible factor (HIF)-1α/Wnt pathway and increasing GATA4/TGF-β expression, thereby blocking IVDD. Our findings provide important insights into the mechanisms through which CESCs ameliorate IVDD and the application of CESCs as a therapy for IVDD.

## Materials and Methods

### Reagents and Antibodies

The Wnt pathway inhibitors MSAB and Wnt agonist 1, the TGF-β agonist SRI-011381, and the TGF-β1 inhibitor pirfenidone (PFD) were obtained from Selleck (Shanghai, China). Antibodies against collagen I, CD9, and CD63 were obtained from Beyotime (Shanghai, China). Antibodies against glyceraldehyde 3-phosphate dehydrogenase (GAPDH), collagen II, GATA4, aggrecan (Acan), tumor susceptibility gene 101 (TSG101), and TGF-β1 were purchased from Proteintech (Wuhan, China). Collagenase II was purchased from Sangon Biotech (Shanghai, China). Antibodies against a disintegrin and metalloproteinase with thrombospondin motifs 5 (ADAMTS5), sirtuin 9 (SOX9), transcription factor 4, and β-catenin were purchased from Bioss (Beijing, China). Antibodies against HIF-1α were obtained from Santa Cruz Biotechnology (Dallas, TX, United States). PKH26 was obtained from Sigma (St. Louis, MO, United States). MSC osteogenic differentiation medium, chondrogenic differentiation medium, and adipogenic differentiation medium were provided by Cyagen (Guangzhou, China).

### Isolation and Identification of CESCs

Cartilage endplate stem cells were isolated from CEPs of 4-week-old male Sprague-Dawley rats. We cleaned and washed CEPs with 0.1 M sterile phosphate-buffered saline (PBS). Then, the CEP tissues were mechanically sliced into pieces and digested with 0.2% type II collagenase for 3 h at 37°C. The suspension was filtered, washed in PBS, and centrifuged at 1,000 rpm for 5 min. Finally, cells were cultured in Dulbecco’s modified Eagle medium (DMEM; cat. no. SH30023.01; HyClone) containing 10% fetal bovine serum (cat. no. A6903FBS-500; Invitrogen, Carlsbad, CA, United States) and 1% penicillin-streptomycin at 5% CO_2_ and 37°C. The culture medium was replaced twice a week, and CESCs from passage 2 or 3 were used in our experiments.

To induce CESC chondrogenic differentiation, the cells were cultured in 6-well plates in chondrogenic differentiation medium (cat. no. MUCMX-9004; Cyagen Biosciences, Guangzhou, China) for up to 21 days. The culture medium was changed every 3 days. For inducing CESC osteogenic differentiation, the CESCs were cultured in 6-well plates in osteogenic differentiation medium (cat. no. MUBMX-90021; Cyagen Biosciences) for up to 21 days, with replacement of the culture medium every 3 days. To induce CESC adipogenic differentiation, CESCs were cultured in 6-well plates in adipogenic differentiation medium for up to 21 days with changes in the culture medium to medium A or medium B (cat. no. MUBMX-90031; Cyagen Biosciences) for 3 days. After culturing in elective induction medium according to the above protocols, Alcian blue, Alizarin red, and Oil red O staining were performed to confirm the differentiation of each type. To detect cell surface markers, a flow cytometer (BD Biosciences, CA, United States) was used to identify CESCs by positive expression of CD90 and CD44 and negative expression of CD45 according to the manufacturer’s instructions.

### Exosome Extraction

Exosomes were extracted by differential ultracentrifugation as previously described (Théry et al., 2006). CESCs were cultured in serum-free DMEM (cat. no. SH30023.01; HyClone) for 2 days at 37°C in 5% CO_2_. The culture supernatants were collected and centrifuged at 300 × *g* for 10 min, 2000 × *g* for 10 min, and 10,000 × *g* for 30 min to remove cells, dead cells, and cellular debris. The supernatant was transferred to clean tubes and centrifuged at 100,000 × *g* for 70 min at 4°C. The supernatant was then removed completely, and the pellet was resuspended in PBS. Next, the sample was filtered through a 0.22-μm filter and centrifuged at 100,000 × *g* for 70 min at 4°C. After removing the supernatant as completely as possible, the exosomes were resuspended in 200 μL PBS.

### Identification of Exosomes and Analysis of Exosome Internalization by CESCs

Exosome morphology was observed using transmission electron microscopy (TEM; Philips, Amsterdam, Netherlands), identified according to the expression of characteristic markers, including TSG101, CD63, and CD9, using western blotting. The purified CESC-exosomes (CESC-Exos) were incubated with PKH26 for 5 min at room temperature. Then, 5% bovine serum albumin (BSA) was added to stop the reaction, and exosomes labeled with membrane dyes were obtained. Next, the PKH26-labeled exosomes were resuspended in DMEM and incubated with CESCs at 37°C for 10 h. CESCs stained with 4-6-diamidino-2-phenylindole (DAPI) were placed under a fluorescence microscope (Olympus, Tokyo, Japan) for observation.

### Wound Scratch Assay and Transwell Migration Assay

For wound scratch assays, CESCs treated with different concentrations of exosomes were seeded onto 6-well plates. After the cells reached 80–90% confluence, a pipette tip was used to inflict a wound. The cells were then washed with PBS to remove debris and floating cells. The wound areas were photographed at 0 and 48 h after scratching using a microscope. For transwell migration assays, transwell chambers were placed in 24-well plates, and treated cell suspensions were then seeded onto the interior chambers. The chambers were removed 24 h after seeding, and cells that had migrated through the membranes were enumerated using a microscope.

### Lentiviral Transfection

Lentivirus overexpressing HIF-1α was purchased from GENECHEM (Shanghai, China), and lentivirus overexpressing GATA4 was purchased from Taitool Bioscience (Shanghai, China). CESCs were seeded into 6-well plates at a density of 1.0 × 10^5^ cells/well, and the next day, cells were infected with the lentivirus (multiplicity of infection: 40) in culture medium containing 6 μg/mL polybrene. The medium was replaced with normal medium 24 h after transfection. We used fluorescence microscopy (Olympus, Tokyo, Japan) to measure the efficiency of transfection 3 days later. Western blotting and polymerase chain reaction (PCR) were also performed to analyze the expression of HIF-1α and GATA4 in transfected cells.

### Quantitative PCR Analysis

Total RNA was isolated with TRIzol reagent according to the manufacturer’s protocol (Aioub et al., 2007). cDNA was synthesized from RNA using an RT reagent kit (Takara, Japan). Then, Quantitative PCR (qPCR) was performed in triplicate to analyze gene expression levels using a Bio-Rad CFX96 qPCR machine (Bio-Rad Laboratories, Hercules, CA, United States) with β*-actin* as a normalization control. The primer sequences are listed in Table 1.

**TABLE 1:** Primers used for qPCR in this study.

**Gene**	**Primer sequence (5′ to 3′)**
GATA4(homo)-F	CGACACCCCAATCTCGATATG
GATA4(homo)-R	GTTGCACAGATAGTGACCCGT
GATA4(rat)-F	AGGGGATTCAAACCAGAAAACG
GATA4(rat)-R	GCTGCTGTGCCCATAGTGAGAT
HIF1α-F	ACCTTCATCGGAAACTCCAAAG
HIF1α-R	CTGTTAGGCTGGGAAAAGTTAGG
SOX9(homo)-F	CTCTGGAGACTTCTGAACGA
SOX9(homo)-R	ACTTGTAATCCGGGTGGTC
SOX9(rat)-F	CATGAACGCCTTCATGGTG
SOX9(rat)-R	CTCTCGTTCAGCAGTCTCC
CCR1-F	CTCATGCAGCATAGGAGGCTT
CCR1-R	ACATGGCATCACCAAAAATCCA
CCR3-F	TCAACTTGGCAATTTCTGACCT
CCR3-R	CAGCATGGACGATAGCCAGG
CCR9-F	CTTCAGCTATGACTCCACTGC
CCR9-R	CAAGGTGCCCACAATGAACA
CXCR3-F	TACCTTGAGGTTAGTGAACGTCA
CXCR3-R	CGCTCTCGTTTTCCCCATAATC
CXCR6-F	GAGTCAGCTCTGTACGATGGG
CXCR6-R	TCCTTGAACTTTAGGAAGCGTTT
TGFbeta -F	CTAAGGCTCGCCAGTCCCC
TGFbeta -R	ATTGCGTTGTTGCGGTCCA
EGF-F	TCCAAACGCCGCAGACTTA
EGF-R	CCTCTTGTTCACCCTTATTACCG
FGF2-F	GCGACCCACACGTCAAACTA
FGF2-R	TCCCTTGATAGACACAACTCCTC
CSF1-F	ATGAGCAGGAGTATTGCCAAGG
CSF1-R	TCCATTCCCAATCATGTGGCTA
PDGFa-F	GAGGAAGCCGAGATACCCC
PDGFa-R	TGCTGTGGATCTGACTTCGAG
Actin-F	GGCTGTATTCCCCTCCATCG
Actin-R	CCAGTTGGTAACAATGCCATGT

### Western Blotting

Cells were harvested, and proteins were extracted using RIPA lysis buffer containing the protease inhibitor phenylmethylsulfonyl fluoride. Protein concentrations were measured using a spectrophotometer (Beckman, Fullerton, CA, United States). Equivalent amounts of protein were separated by sodium dodecyl sulfate polyacrylamide gel electrophoresis and transferred to polyvinylidene fluoride membranes by electroblotting. The membranes were blocked in 5% (w/v) skimmed milk for 1 h at room temperature. After blocking, the membranes were incubated with appropriate primary antibodies overnight at 4°C. Subsequently, the membranes were washed three times with PBS and incubated with the corresponding horseradish peroxidase-conjugated secondary antibodies at room temperature for 1 h. Then, the ECL working solution (Millipore, MO, United States) was added to the membranes. Finally, proteins were visualized and detected using a chemiluminescence system (Bio-Rad Laboratories).

### Immunofluorescence

Cartilage endplate stem cells were fixed with 4% paraformaldehyde for 20 min at room temperature and permeabilized using Triton X-100 solution. The cells were then blocked with goat serum for 40 min and incubated with the appropriate primary antibodies overnight at 4°C. After washing three times with PBS, cells were incubated with the fluorescently labeled secondary antibody for 1 h and then stained with DAPI for 5 min at room temperature. Finally, a fluorescence microscope (Olympus, Tokyo, Japan) was used to capture images of stained CESCs.

### Animal Experiments and Micro-Magnetic Resonance Imaging Analysis

All animal experiments were conducted with the approval of the Medical Ethics Committee of Army Medical University. Twenty 2-month-old Sprague-Dawley rats were obtained from the Model Animal Research Center of the Army Medical University and used for *in vivo* experiments. Rats were randomly divided into four groups: control group (*n* = 5), IVDD group (*n* = 5), IVDD + CESC group (*n* = 5), and IVDD + GATA4-overexpressing CESC group (*n* = 5). The IVDD model was established as described previously (Han et al., 2008). Briefly, after the rats (weighing approximately 250 g each) were anesthetized with 2 mL of 5% (w/v) chloral hydrate, the AF of the rats was punctured using a 21-G needle through the tail skin. The length of the needle into the IVD was approximately 5 mm. Then, we rotated the needles 360° and maintained the needle in the disk for 60 s. In the IVDD + CESC group and IVDD + GATA4-overexpressing CESC group, rats were subjected to intradiscal injection of a CESC suspension (20 μL, 10^5^/mL) or GATA4-overexpressing CESC suspension (20 μL, 10^5^/mL). 4 weeks after injection, Micro-magnetic resonance imaging (MRI) was performed using a 7.0-T animal magnet (Bruker Pharmascan, Germany) to evaluate the signal and structural changes of IVD. Rats were sacrificed after micro-MRI scanning, and the disk and CEP tissues were isolated for immunofluorescence analysis.

### Patient Tissues and Histologic Analysis

Cartilage endplate tissues were obtained from patients who underwent spinal fusion at Xinqiao Hospital of Army Medical University for qPCR, western blot, and immunohistochemical staining. This study was approved by the Medical Ethics Committee of Army Medical University, and all patients signed informed consent for tissue collection. Changes in the CEP on MRI were evaluated by two blinded orthopedic doctors using the Modic Classification of degenerative lumbar endplate (type I, II, III; (Modic et al., 1988)). The patient detail information was shown in Table 2. The tissues were fixed with 4% neutral-buffered formalin and decalcified with 10% ethylenediaminetetraacetic acid. The specimens were then dehydrated and embedded in paraffin for serial sectioning. For immunohistochemical analysis, the sections were autoclaved in 0.01 M citrate buffer at 110°C for 15 min to achieve antigen retrieval. Then, 3% H_2_O_2_ was used to block endogenous peroxidase activity for 10 min, and sections were treated with 5% BSA at 37°C for 30 min to block non-specific binding sites. The sections were incubated with primary antibodies overnight at 4°C. Finally, the sections were incubated with the corresponding horseradish peroxidase-conjugated secondary antibodies for 30 min at room temperature and visualized with 3,30-diaminobenzidine-tetrahy-drochloride. For immunofluorescence analysis, the sections were incubated in 10% hydrogen peroxide/formaldehyde solution at room temperature for 30 min, permeabilized in 0.2% Triton for 5 min, blocked with 5% (w/v) BSA, and incubated with corresponding primary antibodies overnight at 4°C. Then, the sections were incubated with the corresponding fluorescent secondary antibodies and stained with DAPI (Beyotime). Finally, the stained sections were observed using a fluorescence microscope (Olympus).

**TABLE 2:** Patient information in this study.

**CEP tissue no.**	**Age, year**	**Gender**	**Modic classification**
1	45	Female	I
2	52	Female	I
3	40	Male	I
4	55	Female	II
5	58	Male	II
6	43	Male	II
7	62	Male	III
8	49	Female	III
9	56	Female	III

### Statistical Analysis

Quantitative results are described as means ± standard deviations. Statistical data were analyzed by Student’s *t* tests and one-way analysis of variance using GraphPad Prism 7.0 (GraphPad Software Inc., CA, United States). Results with *P* values less than 0.05 were considered statistically significant.

## Results

### Exosomes Promoted the Migration, Invasion, and Transdifferentiation of CESCs Into NPCs via an Autocrine Mechanism

**FIGURE 1:**
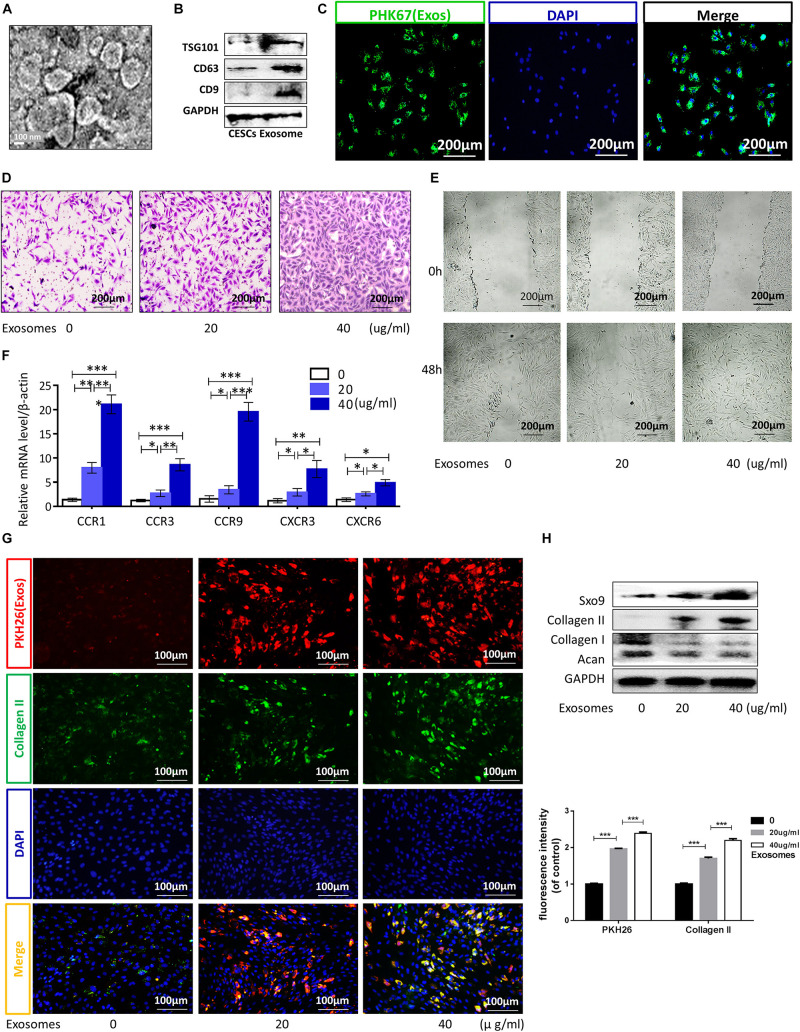
Exosomes promoted CESC migration, invasion, and differentiation into nucleus pulposus cells via autocrine signaling. **(A,B)** TEM images and western blot analyses for identification of exosomes. **(C)** Immunofluorescence of exosomes (green) in CESCs. **(D,E)** Migration and invasion of CESCs treated with 0, 20, or 40 μg/mL exosomes. **(F)** Expression levels of chemokines related to cell migration and invasion in CESCs treated with 0, 20, or 40 μg/mL exosomes. **(G)** Double immunofluorescence of exosomes (red) and collagen II in CESCs. **(H)** Representative western blots and quantification of data for SOX9, collagen II, collagen I, and Acan in CESCs treated with 0, 20, or 40 μg/mL exosomes. All data represent means ± standard deviations. *P* < 0.05 was considered statistically significant. ns: *P* > 0.05; ^∗^*P* < 0.05; ^∗∗^*P* < 0.01; and ***P < 0.001.

Cartilage endplate stem cells were isolated and purified from the CEP of rat tail vertebrae and showed a fibroblast-like morphology with a spindle-like appearance (Supplementary Figure 1A). Low-passage CESCs were induced to differentiate into osteocytes, chondrocytes, and adipocytes in special medium (Supplementary Figure 1B). Flow cytometry showed that stem cell-positive markers (CD90 and CD44) were detected in more than 80% of CESCs, whereas less than 3% of cells expressed the negative marker (CD45; Supplementary Figure 1C). CESC-Exos were collected and purified from CESC culture medium, and the morphology and protein markers of CESC-Exos were identified by TEM and western blotting, respectively. The morphology of CESC is shown in Figure 1A. Western blotting showed that CESC-Exos expressed higher levels of exosomal marker proteins (CD9, CD63, and TSG101) than CESCs (Figure 1B). These results suggested that CESCs could secrete exosomes, similar to other types of stem cells. Exosomes labeled with PKH26 were incubated with CESCs to examine the uptake of exosomes by CESCs. The immunofluorescence results showed that exosomes were dispersed throughout the cytoplasm of CESCs (Figure 1C), indicating that exosomes affected CESCs in an autocrine manner.

Exosomes derived from stem cells promote the differentiation of other cells and may have potential therapeutic applications in some diseases (Zhang et al., 2018; Biswas et al., 2019). To explore the effects of CESC-Exos on CESCs themselves, CESCs were treated with CESC-Exos (20 or 40 μg/mL) at 37°C for 24 h. As shown in Figures 1D,E, treated CESCs exhibited enhanced migratory ability, as demonstrated by increased cell penetration and quicker scratch closure. The qPCR results showed that the activation level of chemokines related to cell migration increased significantly after exosome treatment (Figure 1F). Taken together, autocrine exosomes increase the migration capacity of CESCs. Next, we examined whether CESC-Exos induced CESC differentiation into NPCs. Immunofluorescence showed that the levels of collagen II (an NPC marker protein) were increased following exosome treatment (Figure 1G). Collagen II, SOX9, collagen I, and Acan levels were then measured by western blotting. As expected, the expression of the NPC marker proteins collagen II and SOX9 increased, and that of the negative proteins collagen I and Acan decreased in the treatment group (Figure 1H). Additionally, we found that CESC-Exos could promote the proliferation of NPCs demonstrated by immunofluorescence analysis (Supplementary Figure 2).

### CESC-Exos Facilitated CESC Invasion, Migration, and Differentiation by Activating HIF-1α

**FIGURE 2:**
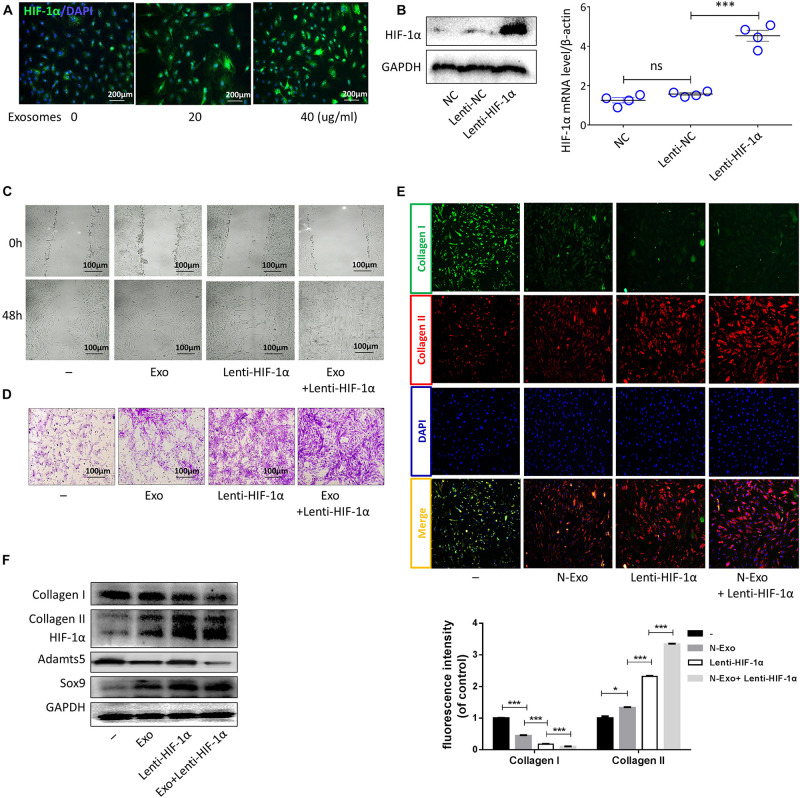
Exosomes promoted CESC invasion, migration, and differentiation by increasing HIF-1α expression. **(A)** HIF-1α expression in CESCs treated with 0, 20, or 40 μg/mL exosomes. **(B)** Representative western blots of HIF-1α and RT-qPCR assays in CESCs treated with NC, Lenti-NC, or Lenti-HIF-1α. **(C,D)** Migration and invasion in CESCs treated with NC, exosomes (40 μg/mL), Lenti-HIF-1α, Lenti-HIF-1α + exosomes (40 μg/mL). **(E)** Double immunofluorescence of collagen I (green) and collagen II (red) in CESCs treated with NC, exosomes (40 μg/mL), Lenti-HIF-1α, or Lenti-HIF-1α + exosomes (40 μg/mL). **(F)** Western blots of collagen I, collagen II, HIF-1α, ADAMTS5, and SOX9 in CESCs treated as described above. All data represent means ± standard deviations. *P* < 0.05 is considered statistically significant. NC: normal control; ns: *P* > 0.05; *P < 0.05 and ***P < 0.001.

The expression level of HIF-1α in NPCs is higher than that in surrounding AF and chondrocytes (Rajpurohit et al., 2002; Risbud et al., 2006). In this study, the protein levels of HIF-1α in cells treated with CESC-Exos were higher than those in CESCs (Figure 2A). Therefore, the effects of HIF-1α in CESCs were tested using lentivirus to overexpress HIF-1α. HIF-1α mRNA and protein levels in the Lenti-HIF-1α group were increased (Figure 2B). As shown in Figures 2C,D, the migration and invasion capacities of CESCs were increased in the HIF-1α group compared with those in the Exo group (40 μg/mL), and these improvements in migration and invasion were even more apparent in the HIF-1α group cotreated with CESCs-Exos (40 μg/mL). Consequently, these data showed that CESC-Exos promoted CESC invasion and migration by increasing HIF-1α expression. Moreover, collagen II increased and collagen I decreased in the HIF-1α group, as demonstrated by immunofluorescence analysis (Figure 2E). Furthermore, treatment of the HIF-1α group with CESC-Exos (40 μg/mL), resulted in a greater increase in collagen II. Compared with exosome treatment, protein expression levels of collagen II and SOX9 increased in HIF-1α-overexpressing cells, whereas the protein expression of collagen I and ADAMTS5 decreased (Figure 2F). These results revealed that CESC-Exos facilitated CESC differentiation by activating HIF-1α signaling.

### HIF-1α Promoted CESC Differentiation Into NPCs by Activating the Wnt/GATA4 Pathway

**FIGURE 3:**
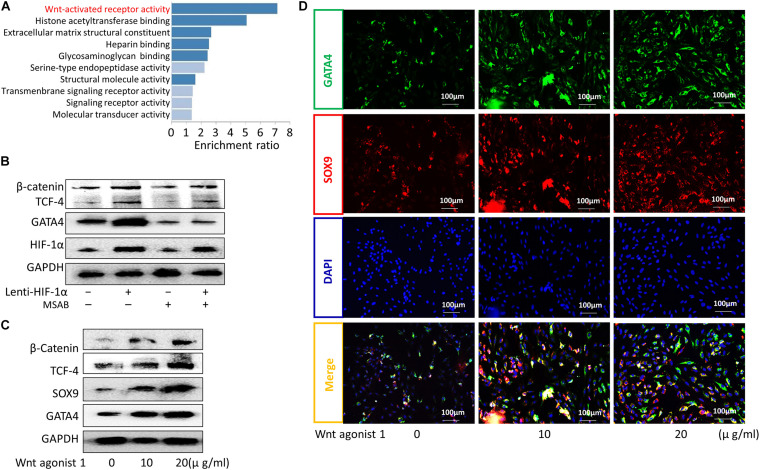
HIF-1α promoted the differentiation of CESCs into NPCs via activation of the Wnt/GATA4 pathway. **(A)** KEGG enrichment analysis of normal and degenerated human cartilage endplate tissue. **(B)** Representative western blots of β-catenin, TCF-4, GATA4, and HIF-1α in CESCs treated with NC, Lenti-HIF-1α, MSAB (inhibitor of the Wnt pathway), or Lenti-HIF-1α + MSAB. **(C)** Western blots of β-catenin, TCF-4, SOX9, and GATA4 in CESCs treated with different concentrations of Wnt agonist 1 (0, 10, or 20 μg/ml). **(D)** Double immunofluorescence of GATA4 (green) and SOX9 (red) in CESCs treated with different concentrations of Wnt agonist 1 (0, 10, or 20 μg/ml).

Hypoxia-inducible factor-1α has been shown to be correlated with the Wnt signaling pathway (Chen et al., 2018; Vallée et al., 2018; Boso et al., 2019), an important pathway modulated by hypoxia (Mazumdar et al., 2010). The GATA4 transcription factor plays key roles in Wnt signaling-regulated processes (Watt et al., 2004; Holtzinger and Evans, 2005), such as differentiation, growth, and survival (Molkentin, 2000; Patient and Mcghee, 2002), in various cell types. In this study, Kyoto Encyclopedia of Genes and Genomes (KEGG) enrichment analyses showed that Wnt signaling was related to CEP degeneration (Figure 3A). Moreover, western blotting showed that the expression of GATA4 increased in the Lenti-HIF-1α group and decreased in the Lenti-HIF-1α + MSAB group (Figure 3B). When CESCs were treated with different concentrations of Wnt agonist 1 (0, 10, or 20 μg/ml), the protein expression levels of β-catenin, TCF-4, SOX9, and GATA4 gradually increased (Figure 3C). Additionally, the protein levels of GATA4 and SOX9 in cells treated with Wnt agonist 1 increased, as demonstrated using immunofluorescence analysis (Figure 3D). These results show that increased HIF-1α enhanced the activation of Wnt signaling, thereby stimulating GATA4 expression to promote CESC differentiation.

### GATA4 Promoted CESC Activation in a TGF-β1-Dependent Manner

**FIGURE 4:**
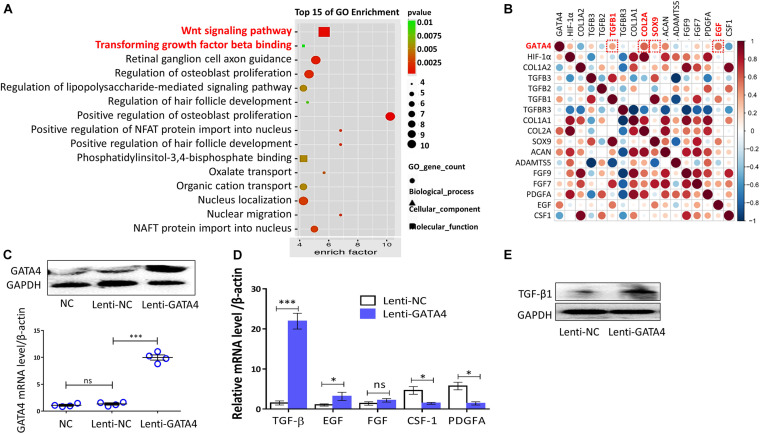
GATA4 significantly increased TGF-β1 expression. **(A)** Gene Ontology (GO) analysis of all differential proteins in normal and degenerated or differentiated human cartilage endplate tissue. **(B)** Correlation analysis of important proteins expressed in human cartilage endplate tissue (GSE153761, https://www.ncbi.nlm.nih.gov/). **(C)** Representative western blots of HIF-1α and RT-qPCR assays in CESCs treated with NC, Lenti-NC, or Lenti-GATA4. **(D)** Expression of *TGF-*β, *EGF*, *FGF*, *CSF-1*, and *PDGFA* in CESCs treated with Lenti-NC or Lenti-GATA4. **(E)** Western blots of TGF-β in CESCs treated with Lenti-NC or Lenti-GATA4. All data represent means ± standard deviations. *P* < 0.05 is considered statistically significant. NC: normal control; ns: *P* > 0.05; ^∗^*P* < 0.05 and ^∗∗∗^*P* < 0.001.

**FIGURE 5:**
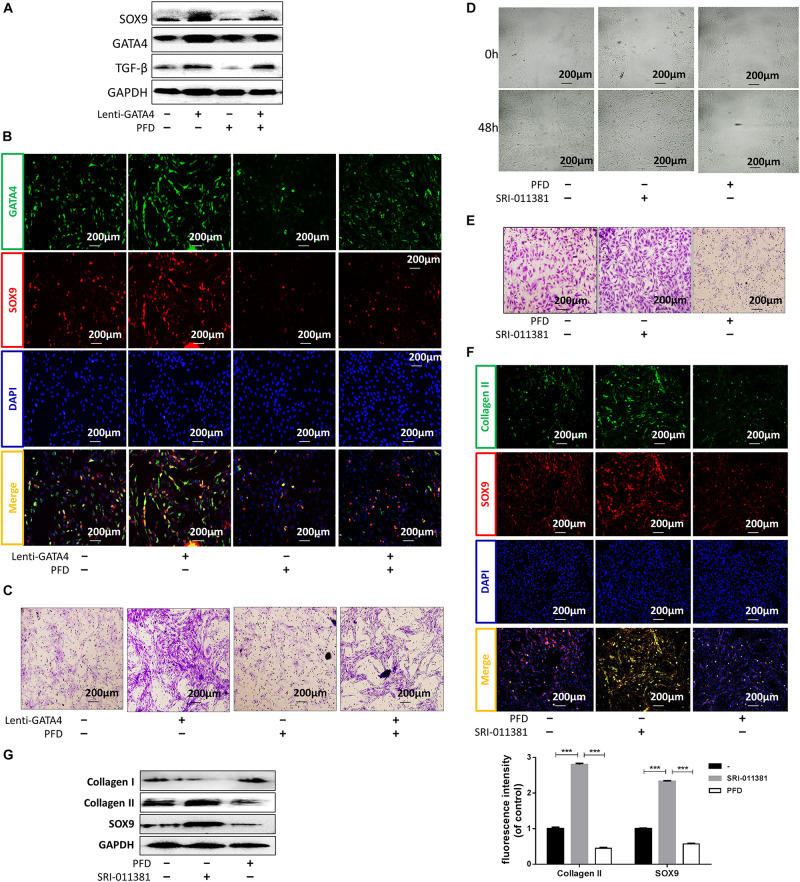
GATA4 promoted CESC invasion, migration, and differentiation via the TGF-β1 signaling pathway. **(A)** Western blots of SOX9, GATA4, and TGF-β in CESCs treated with Lenti-NC or Lenti-GATA4. **(B,C)** Double immunofluorescence of GATA4 (green) and SOX9 (red) and transwell analysis in CESCs treated as described above. **(D,E)** Ability of CESCs to invade and migrate after addition of the TGF-β agonist SRI-011381 or inhibitor pirfenidone (PFD). **(F)** Double immunofluorescence of collagen II (green) and SOX9 (red) in CESCs treated with SRI-011381 or PFD. **(G)** Western blots of collagen I, collagen II, and SOX9 in CESCs treated with NC, SRI-011381, or PFD. All data represent means ± standard deviations. *P* < 0.05 was considered statistically significant. ^∗∗∗^*P* < 0.001.

Gene ontology (GO) and protein correlation analyses were performed to investigate the mechanisms underlying the roles of GATA4 in CESC invasion, migration, and differentiation. GO analysis showed that the Wnt signaling pathway and TGF-β were related to the process of CEP differentiation and degeneration (Figure 4A). As shown in Figure 4B, the protein expression levels of TGF-β1, COL2A, SOX9, and epidermal growth factor (EGF) were closely related to GATA4, as demonstrated using correlation analysis of important proteins. Therefore, we hypothesized that GATA4 may promote CESC activation via the TGF-β1 signaling pathway. To confirm this hypothesis, CESCs were infected with lentiviruses to overexpress GATA4. After transfection, the mRNA and protein levels of GATA4 were increased, as demonstrated by qPCR and western blotting (Figure 4C). qPCR showed that the expression levels of TGF-β1 and EGF increased and that the expression levels of colony stimulating factor-1 (CSF-1) and platelet-derived growth factor (PDGF) decreased in Lenti-GATA4 CESCs (Figure 4D). In addition, the protein level of TGF-β1 was increased in Lenti-GATA4 CESCs, as demonstrated by western blotting (Figure 4E). These results indicated that GATA4 increased the expression of TGF-β1. Moreover, the protein expression of SOX9 and TGF-β1 increased in the Lenti-GATA4 group and decreased in the Lenti-GATA4 group cotreated with the TGF-β inhibitor PFD, indicating that the effects of GATA4 on promotion of CESC differentiation into NPCs was blocked by the TGF-β inhibitor (Figure 5A). Immunofluorescence analysis also indicated that the effects of GATA4 on promoting CESC differentiation into NPCs were blocked by the TGF-β inhibitor (Figure 5B). As shown in Figures 5C–E, the invasion and migration capacities of cells with or without GATA4-expressing lentivirus were blocked by PFD, whereas the TGF-β agonist SRI-011381 improved the invasion and migration capacities of CESCs without treatment. The protein levels of collagen II and SOX9 were increased in CESCs treated with SRI-011381 but decreased in CESCs treated with PFD, as demonstrated by immunofluorescence analysis, indicating that TGF-β promoted CESC differentiation (Figure 5F). The western blotting results were consistent with the observations under fluoroscopy (Figure 5G). Based on the above results, we concluded that GATA4 promoted CESC invasion, migration, and differentiation via TGF-β1 signaling.

### CESCs Overexpressing GATA4 Ameliorated IVDD *in vivo*

**FIGURE 6:**
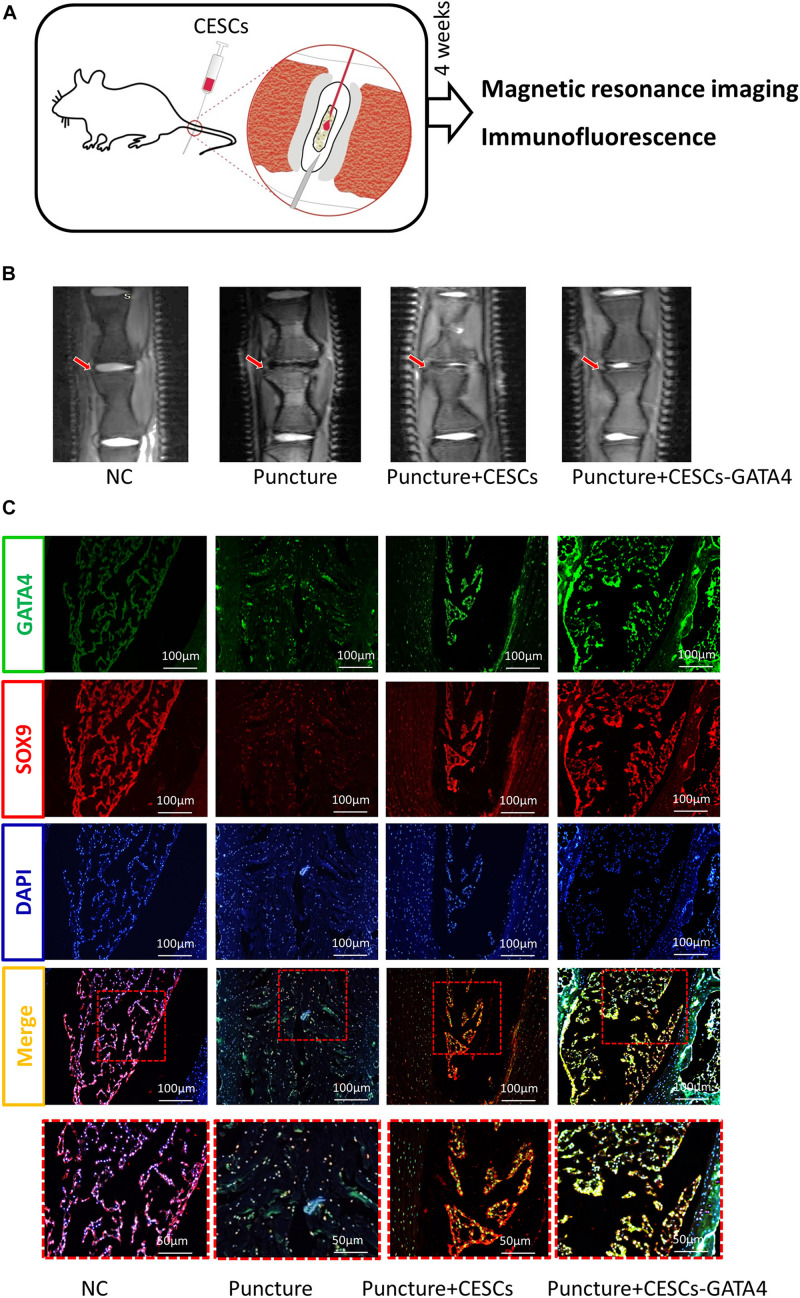
CESCs overexpressing GATA4 enhanced intervertebral disk repair and inhibited intervertebral disk degeneration. **(A)** Experimental steps for CESC treatment via microsyringe in the IVDD model. **(B)** Representative MRI results for rat intervertebral disks treated with NC, puncture, puncture + CESCs (20 μL, 10^5^/mL), or puncture + CESCs overexpressing GATA4 (20 μL, 10^5^/mL). **(C)** Representative double immunofluorescence of SOX9 (red) and cleaved GATA4 (green) images for rat disks in each group (*n* = 5 rats/group).

To further explore the therapeutic effects of CESCs overexpressing GATA4 in IVDD, an IVDD animal model was used in our experiment. Two groups of IVDD rats (*n* = 5 rats/group) were injected with CESCs (20 μL, 10^5^/mL) and CESCs overexpressing GATA4 (20 μL, 10^5^/mL; Figure 6A). IVDD model rats were assessed by micro-MRI at 4 weeks after operation. The MRI results showed that the IVDD of rats in CESCs and CESCs overexpressing GATA4 was less severe and that IVDD was more effectively ameliorated in GAT4-overexpressing CESCs than in CESCs (Figure 6B). Histological immunofluorescence analysis showed that SOX9 and GATA4 were upregulated in CESCs and CESCs overexpressing GATA4 and decreased in the IVDD group without injection; however, the degeneration of the IVD was minimal in CESCs overexpressing GATA4, indicating that CESCs differentiated into NPCs to repair IVDs via GATA4 signaling (Figure 6C). Consequently, CESCs enhanced IVD repair and ameliorated IVDD deterioration *in vivo*.

### CESCs Were Converted Into NPCs in Degenerated and Differentiated CEPs

**FIGURE 7:**
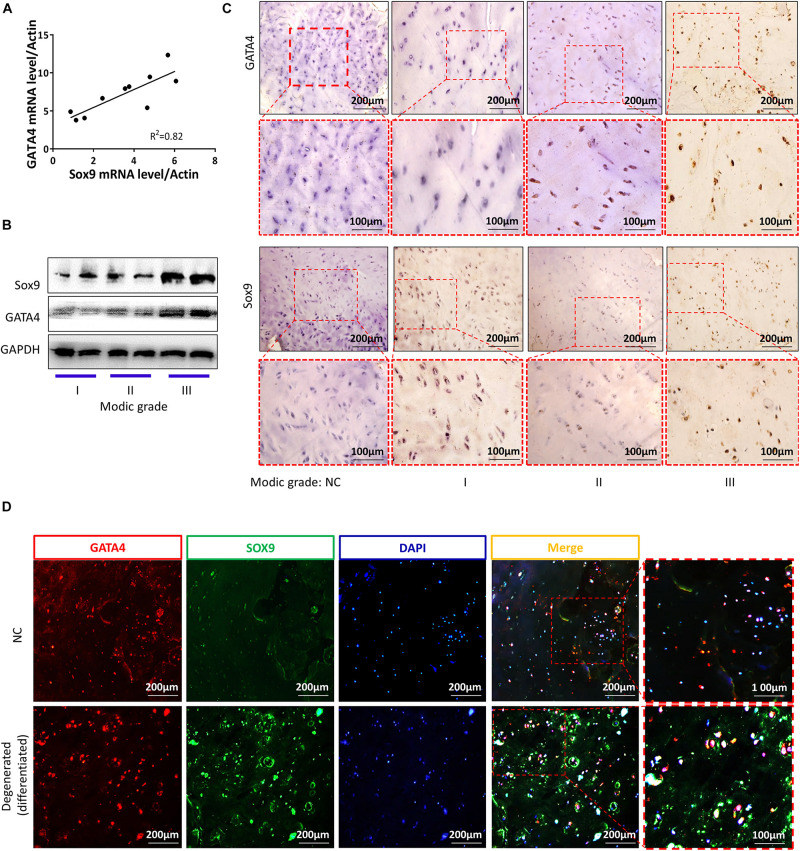
GATA4 and SOX9 levels were increased in degenerated and differentiated cartilage endplates. **(A)** mRNA expression levels of *GATA4* and *SOX9* in cartilage endplates. **(B)** Representative western blots of GATA4 and SOX9 in cartilage endplate inflammation based on Modic grading. **(C)** Immunohistochemical staining of GATA4 and SOX9 in stages 1–3 of cartilage degeneration and differentiation. **(D)** Double immunofluorescence of GATA4 (red) and SOX9 (green) in normal or degenerated and differentiated cartilage endplates.

Cartilage endplates were isolated from the patients. As shown in Figure 7A, there was a clear linear positive correlation between the mRNA levels of *SOX9* and *GATA4* in CEP differentiation and degeneration. The protein levels of SOX9 and GATA4 increased with the development of CEP degeneration, as demonstrated by western blotting (Figure 7B). The results of immunofluorescence and immunohistochemical analyses for SOX9 and GATA4 were similar to those of western blotting (Figures 7C,D). These results suggested that CESCs could be converted into NPCs through the progression of endplate differentiation and degeneration.

## Discussion

Physiotherapy and medicine treatments can relieve the symptoms of LBP in most patients with IVDD, but do not reverse disk degeneration (Lindbäck et al., 2018). Lumbar fusion is the most common choice for severe IVDD; however, adjacent segment degeneration may cause adjacent segment symptoms (Kim et al., 2016; Okuda et al., 2018). Other alternative therapeutic treatments that can repair and regenerate IVD are needed. MSC transplantation has been performed successfully in human degenerated IVD (Noriega et al., 2017). In our study, we found that CESCs could migrate and transdifferentiate into NPCs via autocrine mechanisms. After activation of the HIF-1α/Wnt pathway and increasing GATA4/TGF-β expression, we observed inhibition of IVDD by CESC-Exos.

Exosomes are small vesicles secreted by various types of cells and can establish a potent mode of intercellular communication (Luga et al., 2012). Following their secretion, exosomes can work on their original cells in an autocrine manner, on vicinal cells in a paracrine manner, or on distant cells in an endocrine manner (Milman et al., 2019). After being taken up by cells, exosomes play roles in various cellular activities, including proliferation and differentiation (Barile and Vassalli, 2017; Chang et al., 2018). After treatment with exosomes, we found that CESC migration capacity was significantly improved and that the expression of NPC markers (SOX9 and collagen II) increased. This suggested that CESC-Exos could promote CESC migration and transdifferentiate the CESCs into NPCs in an autocrine manner.

Nucleus pulposus cells live in an avascular and hypoxic niche of the IVD, and the level of HIF-1α in NPCs is increased compared with that in the surrounding cells (Rajpurohit et al., 2002; Risbud et al., 2006). HIF-1α is a transcription factor that is important for maintaining NPC matrix synthesis and regulating glycolytic metabolism (Risbud et al., 2006; Agrawal et al., 2007, 2008). Additionally, HIF-1α is necessary for postnatal NPC survival and to maintain intracellular pH homeostasis by regulating carbonic anhydrases 9 and 12 (Merceron et al., 2014; Silagi et al., 2018). Some growth factors, such as insulin-like growth factor-2 and transforming growth factor-α, are HIF-1α target genes (Feldser et al., 1999; Krishnamachary et al., 2003). These factors activate signal transduction pathways that lead to cell proliferation by binding to their cognate receptors (Semenza, 2003). Furthermore, previous studies suggested that HIF-1α was associated with tumor metastasis, promoting cell migration and invasion (Liu et al., 2016; Hu et al., 2020). Our immunofluorescence results revealed that the expression level of HIF-1α increased in CESCs after treatment with exosomes, indicating that HIF-1α played important roles in CESC transdifferentiation. Subsequently, overexpression of HIF-1α CESCs was used in our experiments to explore the underlying mechanisms of CESC transdifferentiation. Immunofluorescent staining, western blotting, wound scratch assays, and transwell migration assays showed that exosomes promoted CESC migration and differentiation by increasing HIF-1α expression. The Wnt/β-catenin pathway is an important pathway modulated by hypoxia (Giles et al., 2006; Mazumdar et al., 2010). Based on our KEGG enrichment analysis, we suspected that the increase in HIF-1α activated the Wnt/β-catenin pathway to react with CESCs. Therefore, MSAB and Wnt agonist 1 were used to treat HIF-1α-overexpressing CESCs and CESCs, respectively. As expected, we verified that increasing HIF-1α expression activated the Wnt pathway to promote the migration and differentiation of CESCs into NPCs.

GATA-binding protein 4 was first discovered in cardiac tissue and was shown to regulate heart development (Kelley et al., 1993). GATA4 is a member of the highly conserved zinc-finger transcription factor family and plays key roles in regulating cell differentiation, growth, and survival in addition to cardiac cells (Molkentin, 2000; Patient and Mcghee, 2002). Some studies have demonstrated that Wnt/β-catenin signaling regulates the expression of GATA4 at the transcriptional level (Watt et al., 2004; Holtzinger and Evans, 2005). Consistent with previous studies, our western blotting and immunofluorescence analyses showed that GATA4 was regulated by the Wnt signaling pathway. We concluded that increased HIF-1α enhanced the activation of Wnt signaling and upregulated GATA4 expression to promote CESC differentiation. According to GO and protein correlation analyses, we found that GATA4 may be related to the TGF-β1 signaling pathways. To confirm the relationship between GATA4 and TGF-β1, CESCs overexpressing GATA4 as well as PFD and SRI-011381 were used in our experiments. Although TGF-β1 and EGF are both related to GATA4 signaling, qPCR indicated that the expression of TGF-β1 increased more significantly compared with the expression of EGF. Finally, our results demonstrated that GATA4 promoted CESC migration and differentiation by increasing TGF-β1 expression.

We further examined the roles of CESCs in a rat model of IVDD. Consistent with our previous *in vitro* study, our current findings showed that CESCs could ameliorate IVDD deterioration *in vivo* and that the degree of IVDD was minimal in CESCs overexpressing GATA4. This suggested that CESCs may promote transdifferentiation and effectively ameliorate IVDD via GATA4 signaling. IVDD is accompanied by CEP degeneration, and CEP inflammation is a characteristic of IVDD. Our results suggested that the CESCs were gradually converted into NPCs with the development of CEP degeneration in humans.

In conclusion, we demonstrated that the HIF-1α/Wnt signaling pathway in CESCs was activated by autocrine exosomes to increase the expression of GATA4 and TGF-β1, thereby promoting the migration of CESCs into the IVD and the transformation of CESCs into NPCs and inhibiting IVDD. Our findings provide insights into the potential applications of CESCs in the treatment of IVDD.

## Data Availability Statement

The original contributions presented in the study are included in the article/Supplementary Material, further inquiries can be directed to the corresponding author/s.

## Ethics Statement

The Ethics Committee of the Xinqiao Hospital of Army Medical University approved the present study (AF/SC-08/1.0). The patients/participants provided their written informed consent to participate in this study. The animal study was reviewed and approved by The Animal Ethics Committee Army Medical University approved all studies [No. SYXK(yu)2017-0002].

## Author Contributions

LL and JG: conception and design, conducting experiments, collection and/or assembly of data, data analysis, and interpretation manuscript writing. HZ, YT, JQ, and JZ: provision of study material and data analysis. YaZ, JC, and CL: conducting experiments and animal modeling assistance. YuZ, ZT, YL, and ML: revising the manuscript, administrative support, and financial support. All authors contributed to the article and approved the submitted version.

## Conflict of Interest

The authors declare that the research was conducted in the absence of any commercial or financial relationships that could be construed as a potential conflict of interest.

## Supplementary Material

Supplementary Figure 1 Extraction and identification of CESCs derived from rat CEP. **(A)** Morphology of P1 and P3 CESCs at 80% confluence and P5 CESCs at 100% confluence. **(B)** Oil red O, Alcian blue, and Alizarin red S staining analysis of stem cell characteristics after adipogenic, chondrogenic, and osteogenic induction. **(C)** Flow cytometry analysis of stem cell-positive and -negative markers CD90, CD44, and CD45 in CESCs. The blue curves represent the fluorescence intensity of unstained cells, and the red curves indicate the fluorescence intensity of CESCs stained with the corresponding antibodies.

Supplementary Figure 2 Exosomes promote the proliferation of NPCs. Double immunofluorescence of EdU (green) and PKH26 (red) in NPCs treated with different concentrations of exosomes (0, 20, or 40 μg/ml).
